# Lycorine hydrochloride suppresses stress‐induced premature cellular senescence by stabilizing the genome of human cells

**DOI:** 10.1111/acel.13307

**Published:** 2021-01-17

**Authors:** Weina Zhang, Jiaqing Yang, Yu Chen, Renhao Xue, Zhiyong Mao, Wen Lu, Ying Jiang

**Affiliations:** ^1^ Clinical and Translational Research Center of Shanghai First Maternity & Infant Hospital Shanghai Key Laboratory of Signaling and Disease Research Frontier Science Center for Stem Cell Research School of Life Sciences and Technology Tongji University Shanghai China; ^2^ Department of Gynecology of Shanghai First Maternity & Infant Hospital Tongji University School of Medicine Shanghai China

**Keywords:** cellular senescence, genome integrity, homologous recombination, Lycorine hydrochloride, nonhomologous end joining, SIRT1, SIRT6

## Abstract

Lycorine, a natural compound isolated from the traditional Chinese medicinal herb *Lycoris radiata*, exhibits multiple pharmacological effects, such as anti‐inflammatory, antiviral, and anticancer effects. Accumulating evidence also indicates that lycorine might hold the potential to treat age‐associated Alzheimer's disease. However, whether lycorine is involved in delaying the onset of cellular senescence and its underlying mechanisms has not been determined. Here, we demonstrate that the salt of lycorine, lycorine hydrochloride, significantly suppressed stress‐induced premature cellular senescence (SIPS) by ~2‐fold, as determined by senescence‐associated beta‐galactosidase (SA‐β‐gal) staining and the expression of p16 and p21. In addition, pretreating cells with lycorine hydrochloride significantly inhibited the expression of CXCL1 and IL1α, two factors of the senescence‐associated secreted phenotype (SASP) in SIPS cells. Further experiments revealed that lycorine hydrochloride promoted both the homologous recombination (HR) and nonhomologous end joining (NHEJ) pathways of DNA double‐strand break (DSB) repair. Mechanistic studies suggested that lycorine hydrochloride treatment promoted the transcription of SIRT1 and SIRT6, critical longevity genes positively regulating both HR and NHEJ repair pathways, thereby stimulating DSB repair and stabilizing genomes. Inhibiting SIRT1 enzymatic activity abrogated the protective effect of lycorine hydrochloride on delaying the onset of SIPS, repairing DSBs, and restoring genome integrity. In summary, our work indicates that lycorine hydrochloride might hold therapeutic potential for treating age‐associated diseases or promoting healthy aging by stabilizing genomes.

## Graphical abstract

Lycorine hydrochloride delays stress‐induced premature cellular senescence by promoting DSB repair.

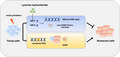

## 1 INTRODUCTION

Lycorine is a pharmacologically active alkaloid widespread in Amaryllidaceae family. Mounting evidence indicates diverse pharmacological functions of plant‐derived lycorine, such as antiviral (Renard‐Nozaki et al., 1989; Szlavik et al., 2004), antibacterial (Tan et al., 2011), antiparasitic (Cedron et al., 2010), anti‐inflammatory (Kang et al., 2012; Wang et al., 2018), and antitumor effects (Lamoral‐Theys et al., 2009; Ying et al., 2017). The molecular mechanisms of multiple functions of lycorine include the induction of apoptosis (Li et al., 2007; Liu et al., 2004), inhibition of cell cycle progression (Li et al., 2012; Liu et al., 2010), and autophagy (Roy et al., 2016). Lycorine hydrochloride, as a main active component of the medicinal herb *Lycoris radiata*, has similar properties to lycorine, including antitumor effects. Another critical feature of lycorine and lycorine hydrochloride is low toxicity to normal cells (Cao et al., 2013).

Amaryllidaceae family plants have been used as therapeutic agents against central nervous system (CNS)‐related maladies such as age‐associated Alzheimer's disease (AD) (Adams et al., 2007), which is characterized by impaired cognitive function and decreased memory capacity. Moreover, aging is another risk factor for AD, and senescent cells irreversibly lose the ability to divide, thus accumulating in tissues *in vivo* with age (Campisi et al., 2011) and secrete numerous proteases, cytokines and growth factors that lead to the senescence‐associated secretory phenotype (SASP) (Sun et al., 2018). Interleukin 1α (IL1α) (Kuilman et al., 2008) and CXCL1 (Kim et al., 2018), two key components of the SASP, reinforce senescence growth arrest in neighboring cells, which may contribute to age‐related declines in organ function, while inflammation and pathologies driven by the increase in senescence cells during aging cause age‐associated diseases (Jeyapalan & Sedivy, 2008).

Normal human diploid cells undergo replicative senescence as a result of telomere shortening, while cells exposed to stress stimuli, such as ionizing radiation (IR) before reaching the Hayflick limit exhibit the same phenotype as replicative senescent cells; this process is called stress‐induced premature cellular senescence (SIPS) (Suzuki & Boothman, 2008). Interestingly, SIPS is usually induced by genotoxic stresses that cause various types of DNA damage (Chen et al., 2007). Among all types of DNA damage, double‐strand breaks (DSBs) are the most deleterious. If DSBs are not repaired or are inappropriately repaired, severe consequences such as SIPS can result (Lombard et al., 2005; Torgovnick & Schumacher, 2015). Nonhomologous end joining (NHEJ) and homologous recombination (HR) repair are two competitive pathways for mending broken DNA ends. NHEJ is active throughout the cell cycle, while HR is preferentially initiated in the S and G2 phases when a sister chromatid is available to serve as a template (Ceccaldi et al., 2016). In addition to the cell cycle stage, the initiation of these two pathways is also determined by BRCA1/CtIP and 53BP1/Rif1, which compete for the DNA end excision step (Bunting et al., 2010). For HR, the MRN complex and CtIP participate in DNA end excision to form single‐strand DNA (ssDNA), and the initiation of HR follows. RPA2 binds to the resected end to prevent ssDNA from degradation, which is subsequently replaced by RAD51 to enable a match with an available sister chromatid (San Filippo et al., 2008). In contrast, NHEJ repairs the broken ends by ligation, concomitantly with the error‐prone mutation. Major factors involved in NHEJ repair include KU70, KU80, and DNA‐PKcs, which are the subunits of the trimer complex DNA‐PK. Downstream factors recruited by DNA‐PK are Artemis, XRCC4, XLF, and DNA ligase 4 (Pannunzio et al., 2018).

Here, we found that lycorine hydrochloride inhibited the onset of SIPS in a dose‐dependent manner and attenuated the expression of specific SASP factors, as indicated by the senescence‐associated beta‐galactosidase (SA‐β‐gal) activity. Moreover, using our previously established reporter cassettes for the analysis of NHEJ and HR repair efficiency, we found that lycorine hydrochloride promoted DSB repair by both NHEJ and HR. Further mechanistic studies indicated that lycorine hydrochloride accelerated the clearance of **γ**H2AX and 53BP1 foci and stimulated the recruitment of RPA2 to DSB sites without changing the expression levels of NHEJ‐ and HR‐related factors. Interestingly, treating cells with lycorine hydrochloride enhanced genome integrity. Furthermore, our findings revealed that lycorine hydrochloride also regulates genomic stability and SA‐β‐gal activity by stimulating the expression of SIRT1, thereby further inhibiting the onset of SIPS.

## 2 RESULTS

### 2.1 Lycorine hydrochloride suppresses the cellular senescence induced by IR

**FIGURE 1:**
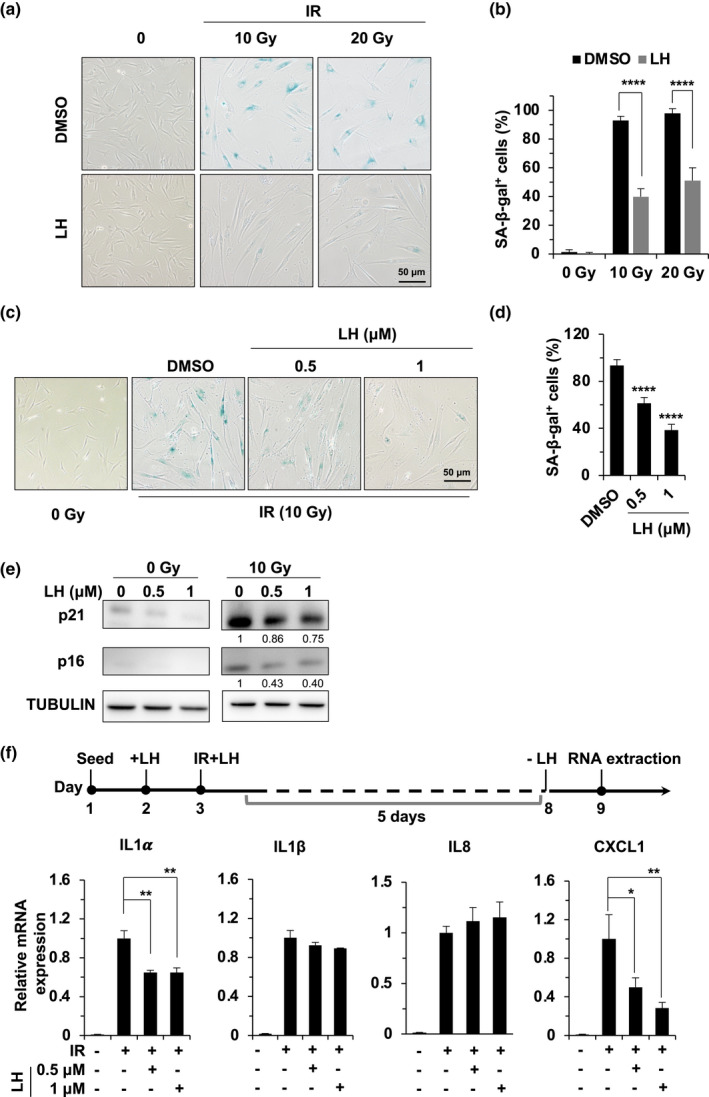
Lycorine hydrochloride inhibits IR‐induced cellular senescence. (a, b) HCA2 fibroblasts exposed to X‐ray irradiation at the indicated doses (10 and 20 Gy) were treated with DMSO or lycorine hydrochloride and incubated for 14 days, followed by SA‐β‐gal staining. The concentration of lycorine hydrochloride was 1 μM. Representative images of SA‐β‐gal staining are shown in (a), and the percentage of SA‐β‐gal‐positive cells is shown in (b). (c,d) Cells were incubated with lycorine hydrochloride at the indicated concentrations after X‐ray irradiation at 10 Gy. Cellular senescence was assessed by SA‐β‐gal staining (blue)in (c), and the quantitative analysis of SA‐β‐gal‐positive cells is shown in (d). (e) HCA2 cells were treated with the indicated concentration of lycorine hydrochloride for 6 days with or without IR, and the protein expression of p16 and p21 was analyzed by Western blotting. Tubulin served as a loading control. (f) Lycorine hydrochloride treatment suppresses the expression of SASP. The scheme of the experimental design is shown in the upper panel. HCA2 fibroblasts were pretreated with lycorine hydrochloride for 1 day, followed by X‐ray irradiation at 10 Gy. The cells were then harvested on day 6 post‐IR. Relative mRNA expression of the indicated SASP factors was analyzed by qRT‐PCR. Error bars represent SD. *p < 0.05, **p < 0.01, ****p < 0.001, *t* test. All experiments were repeated at least three times. LH, lycorine hydrochloride.

Since lycorine has the potential to ameliorate AD pathology and because removing senescent cells in the brain prevents the cognitive decline associated with AD (Elgorashi et al., 2004; Lopez et al., 2002), we hypothesized that lycorine hydrochloride might suppress the induction of SIPS. Therefore, we set out to test whether lycorine hydrochloride pretreatment inhibits IR‐induced SIPS. We pretreated human diploid HCA2 foreskin fibroblasts with lycorine hydrochloride at a concentration of 1 μM, irradiated them with X‐rays at doses of 10 and 20 Gy, cultured them for 14 days and then stained them with β‐gal. We found that ~92.8% (10 Gy) and ~97.8% (20 Gy) of these HCA2 cells without lycorine hydrochloride pretreatment were positive for β‐gal, while only ~39.9% (10 Gy) and ~51.1% (20 Gy) of the lycorine hydrochloride‐treated cells were β‐gal‐positive (Figure 1a,b). We also observed that the suppressive effect of lycorine hydrochloride on SIPS was dose‐dependent (Figure 1c,d; Figure S1a,b).

To validate these results, we examined the expression levels of p16^INK4A^ and p21^CIP1/WAF1^, two CDK inhibitors and critical biomarkers of cellular senescence (Brown et al., 1997; Coppe et al., 2011; Stein et al., 1999). In agreement with the β‐gal staining assay results, the expression of these two proteins decreased in a lycorine hydrochloride dose‐dependent fashion (Figure 1e).

Consistent with previous reports (Herranz et al., 2015), the expression of SASP genes, including IL1α and CXCL1, was stimulated in the control SIPS cells, while in cells treated with lycorine hydrochloride, the expression of these two SASP factors was significantly suppressed (Figure 1f). We examined whether treating SIPS cells with lycorine hydrochloride can inhibit the expression of these two SASP genes. We first subjected SIPS cells with X‐rays and then treated them with lycorine hydrochloride for two days before harvesting them for the analysis of SASP‐related gene expression using quantitative PCR. The results indicated that lycorine hydrochloride impaired the expression of the SASP in senescent cells. (Figure S2).

Taken together, our results show that lycorine hydrochloride suppresses the onset of SIPS and the expression of these two SASP‐related genes.

### 2.2 Lycorine hydrochloride accelerates the repair of damaged DNA and promotes genome stability

**FIGURE 2:**
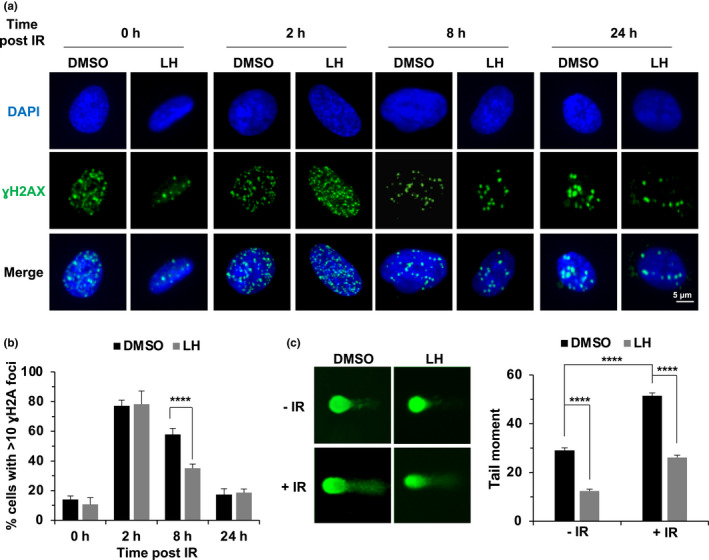
Lycorine hydrochloride accelerates the clearance of γH2AX foci and maintains genome integrity. (a,b) Lycorine hydrochloride accelerates the clearance of γH2AX foci. HCA2 cells were treated with 2 Gy of X‐ray radiation, incubated with lycorine hydrochloride at a concentration of 1 μM, and then immunostained for γH2AX foci at the indicated time points. Representative images are shown in (a), and the quantification of cells with >10 γH2AX foci is shown in (b). (c) Lycorine hydrochloride promotes the genomic stability of HCA2 cells. Representative images of the analysis of genomic stability of the HCA2 cells treated with 1 μM lycorine hydrochloride for 24 h (left panel) based on a comet assay. The tail moment of at least 50 cells for each group was quantified using Comet Score software (Sumerduck, VA, USA) (right panel). Error bars in (b) represent the SEM. Error bars in (c) represent the SEM. *p* < 0.001, *t* test. All experiments were repeated at least three times. LH, lycorine hydrochloride.

Since X‐rays induced the same amounts of DNA damage in both control and lycorine hydrochloride‐treated cells, we hypothesized that lycorine hydrochloride might promote DNA repair to suppress the onset of SIPS. Therefore, we set out to examine the kinetics of γH2AX and 53BP1 foci elimination in cells treated with lycorine hydrochloride and exposed to X‐rays. We found that lycorine hydrochloride did not affect the formation of γH2AX and the recruitment of 53BP1, as the foci numbers of γH2AX and 53BP1 were not affected by lycorine hydrochloride treatment 2 h after X‐ray irradiation, while we observed an ~2‐fold reduction in γH2AX foci number in lycorine hydrochloride‐treated cells 8 h post‐IR (Figure 2a,b) and an ~33% reduction in the number of 53BP1 foci positive 16 h post‐IR with lycorine hydrochloride treatment (Figure S3a,b), indicating that lycorine hydrochloride promotes the repair of X‐ray‐induced DNA damage.

To further examine the influence of lycorine hydrochloride on genomic stability, an alkaline comet assay was performed. We found that lycorine hydrochloride treatment significantly inhibited genomic instability by ~2‐fold in both the unirradiated and X‐ray‐irradiated cells, as measured by tail moment (Figure 2c).

In summary, lycorine hydrochloride accelerates the repair of damaged DNA and promotes genomic stability.

### 2.3 Lycorine hydrochloride elevates DSB repair efficiency by both NHEJ and HR without impacting the expression of DNA repair factors

**FIGURE 3:**
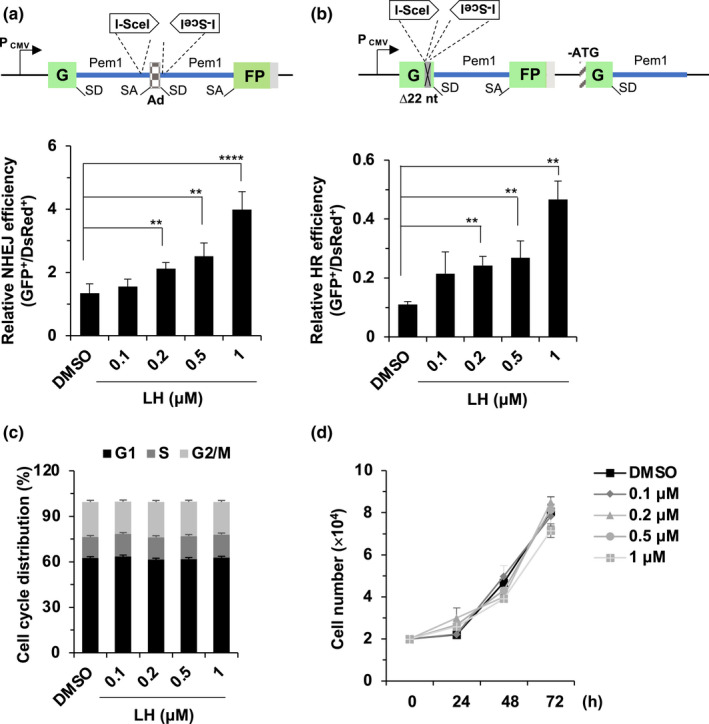
The effects of lycorine hydrochloride treatment on DSB repair efficiency and cell proliferation. (a) Lycorine hydrochloride promotes NHEJ repair efficiency in HCA2‐I7c cells harboring the NHEJ reporter. (b) Lycorine hydrochloride promotes HR efficiency in HCA2‐H15c cells harboring the HR reporter. The NHEJ and HR reporters were constructed as described previously (Mao et al., 2008). Both NHEJ and HR reporters are based on the GFP gene. The NHEJ reporter contains two GFP exons and an adenoviral exon (AD2) separated by a rat Pem1 intron. Two I‐SceI recognition sites are inserted before and after the Ad2 exon. SD and SA represent splice donor and splice acceptor, respectively. In the absence of the I‐SceI restriction enzyme, the Ad2 exon is spliced into the GFP gene to interrupt its expression, leading to lack of GFP expression. In contrast, the induction of DSBs by I‐SceI digestion removes the Ad2 exon. Successful NHEJ transfection can reconstitute GFP expression, rendering cells GFP^+^. The HR reporter contains two inactivated GFP genes. In the first copy, 22 nt are replaced with two I‐SceI regeneration sites in the first copy of GFP to interrupt GFP expression. The second copy lacks a second exon of GFP and the start codon. Only successful HR can reconstitute the GFP expression of the I‐SceI‐digested HR reporter. (c) Lycorine hydrochloride had no influence on cell cycle distribution. HCA2 cells were incubated with lycorine hydrochloride at different concentrations for 48 h. Cell cycle distributions were examined by flow cytometry after PI staining. (d) The effect of lycorine hydrochloride treatment on cell proliferation. HCA2 fibroblasts were treated with lycorine hydrochloride at the indicated concentration, and cells were counted every 24 h. Error bars represent SD. **p < 0.01, ****p < 0.001, *t* test. All experiments were repeated at least three times. LH, lycorine hydrochloride.

The major types of DNA damage induced by X‐ray irradiation are DSBs (Goodhead, 1994). Since lycorine hydrochloride accelerates the repair of DNA damaged by X‐ray‐induced, it is very possible that lycorine hydrochloride promotes DSB repair by NHEJ and/or HR. To test this hypothesis, we employed our well‐established reporter cell lines to measure the efficiency of NHEJ and HR repair (Seluanov et al., 2010) (Figure 3a,b). These cell lines harbor one copy of an integrated GFP‐based reporter for quantifying NHEJ or HR repair. Along with the vector encoding I‐SceI, we also included the DsRed vector to normalize the transfection efficiency. Using these reporter lines, we found that lycorine hydrochloride promoted both NHEJ and HR repair in a dose‐dependent manner (Figure 3a,b), demonstrating that lycorine hydrochloride maintains genome integrity by promoting NHEJ and HR repair.

The preference for these two pathways is greatly dependent on the cell cycle stage when the DSB repair occurs (Ceccaldi et al., 2016; Essers et al., 2002; Symington & Gautier, 2011). We then sought to determine whether lycorine hydrochloride affected the cell cycle distribution. Propidium iodide (PI) staining experiments demonstrated that lycorine hydrochloride did not have any effect on cell cycle distribution (Figure 3c), indicating that the lycorine hydrochloride‐mediated increase in HR and NHEJ repair was not a secondary effect of cell cycle arrest.

Consistently, we did not observe any significant change in cell number (Figure 3d) or apoptosis rates as assayed by Annexin‐V staining (Figure S4a,b), indicating low toxicity of lycorine hydrochloride.

To elucidate the regulatory mechanisms of lycorine hydrochloride on NHEJ and HR repair, we first examined the expression levels of important proteins participating in NHEJ and HR repair. We did not observe any obvious effect of lycorine hydrochloride on the expression of the analyzed factors involved in NHEJ, namely, 53BP1, DNA‐PKcs, KU70, KU80, XRCC4, LIG4, and XLF, or HR factors, including MRE11, RAD50, NBS1, BRCA1, PARP1, CtIP, EXO1, RPA2, and RAD51 (Figure S5a,b).

### 2.4 Lycorine hydrochloride affects DNA repair by promoting the expression of SIRT1 at the transcriptional level

**FIGURE 4:**
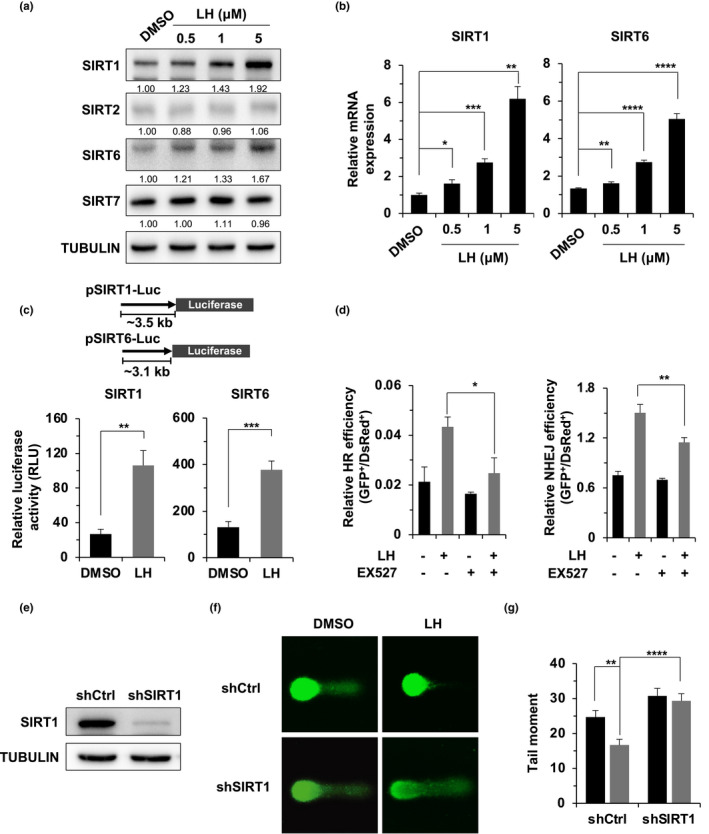
The enhancement of DSB repair and genomic stability by lycorine hydrochloride is dependent on SIRT1. (a) Lycorine hydrochloride stimulated the protein expression of SIRT1 and SIRT6. HCA2 fibroblasts were treated with the indicated concentrations of lycorine hydrochloride for 48 h, and the expression levels of SIRT1, SIRT2, SIRT6, and SIRT7 were analyzed by Western blotting. (b) Lycorine hydrochloride promoted the mRNA expression levels of SIRT1 and SIRT6. HCA2 cells were treated with lycorine hydrochloride at the indicated concentrations for 48 h, and the cells were collected for mRNA expression analysis with quantitative RT‐PCR. (c) Lycorine hydrochloride promotes the mRNA expression levels of SIRT1 and SIRT6 by activating promoter activity. HCA2 cells were treated with lycorine hydrochloride at a concentration of 1 μM one day before transfection with the luciferase reporter or pEGFP‐N1. Relative luciferase activity was measured by the ratio of luciferase activity versus the percentage of GFP^+^ cells. (d) EX527 partially abolished the lycorine hydrochloride‐mediated stimulation of DSB repair by HR and NHEJ. (e) Western blot analysis of HCA2‐hTERT cells with a stably integrated shRNA vector against the SIRT1 gene to verify SIRT1 depletion. (f) Representative images of the alkaline comet assay used for the analysis of the genomic stability of the SIRT1‐depleted cells, which were treated with 1 μM lycorine hydrochloride for 24 h before the comet assay. (g) Quantification of the tail moment from at least 50 cells. Error bars represent SD. *p < 0.05, **p < 0.01, ***p < 0.005, ****p < 0.001, *t* test. All experiments were repeated at least three times. LH, lycorine hydrochloride.

To further examine how lycorine hydrochloride promotes NHEJ and HR, we hypothesized that lycorine hydrochloride might activate upstream factors participating in DSB repair. Several members of the SIRTUIN family have been demonstrated to participate in both NHEJ and HR repair through a number of different mechanisms (Chen et al., 2017, 2019; Lin et al., 2015; Mao et al., 2011). We then analyzed whether lycorine hydrochloride treatment can potentially affect the expression of the four nuclear‐localized sirtuins SIRT1, SIRT2, SIRT6, and SIRT7. We found that the expression of SIRT1 and SIRT6 was enhanced by lycorine hydrochloride in a dose‐dependent manner (Figure 4a). Quantitative PCR analysis indicated that the lycorine hydrochloride‐mediated stimulation of SIRT1 and SIRT6 protein levels was the result of an increase at the transcriptional level (Figure 4b). Additional luciferase assays demonstrated that lycorine hydrochloride enhanced SIRT1 and SIRT6 promoter activity, thereby stimulating SIRT1 and SIRT6 mRNA levels (Figure 4c).

Both SIRT1 and SIRT6 play roles in DSB repair by HR and NHEJ (Chen et al., 2019; Jeong et al., 2007; Lin et al., 2015; Mao et al., 2011), but we observed a more robust stimulatory effect of lycorine on SIRT1 expression (Figure 4a,b). We therefore focused our study on SIRT1. SIRT1 has been suggested to promote HR by deacetylating BRG1 to relax the chromatin around damaged DNA, thereby accelerating the recruitment of end resection factors such as RPA2 (Chen et al., 2019). We examined the kinetics of the focal clearance of RPA2, the critical single‐strand DNA‐binding protein involved in the end resection step of HR. We found that lycorine hydrochloride treatment significantly promoted the recruitment of RPA2 to damaged DNA sites (Figure S5c,d), indicating that the promotion of DSB repair by lycorine hydrochloride is probably mediated by SIRT1.

### 2.5 SIRT1 depletion abolishes the lycorine hydrochloride‐mediated effects on DNA repair, genomic stability, and the onset of SIPS

We then performed further experiments to test whether SIRT1 enzyme activity regulates the lycorine hydrochloride‐mediated promotion of DSB repair. We found that treatment with EX527, a SIRT1 inhibitor, completely abolished the lycorine hydrochloride‐mediated stimulation of HR repair. In contrast, although EX527 significantly suppressed the lycorine hydrochloride‐mediated stimulation of NHEJ repair, the effect was rather modest (Figure 4d). We proposed that SIRT6 might play roles in the lycorine hydrochloride‐mediated stimulation of NHEJ repair, as we observed a mild increase in SIRT6 levels in lycorine hydrochloride‐treated cells. Indeed, depleting SIRT6 impaired the effect of lycorine hydrochloride on NHEJ repair (Figure S6a). Consistently, blocking the enzymatic activities of SIRT1 and SIRT6 with nicotinamide (NAM) inhibited the stimulatory effect of lycorine hydrochloride on both HR and NHEJ repair (Figure S6b).

Additionally, comet assays also indicated that SIRT1 depletion or enzyme activity blockade abolished the lycorine hydrochloride‐mediated reduction in the tail moment (Figure 4e–g; Figure S7a,b), suggesting that the lycorine hydrochloride‐mediated maintenance of genome integrity is dependent on SIRT1.

**FIGURE 5:**
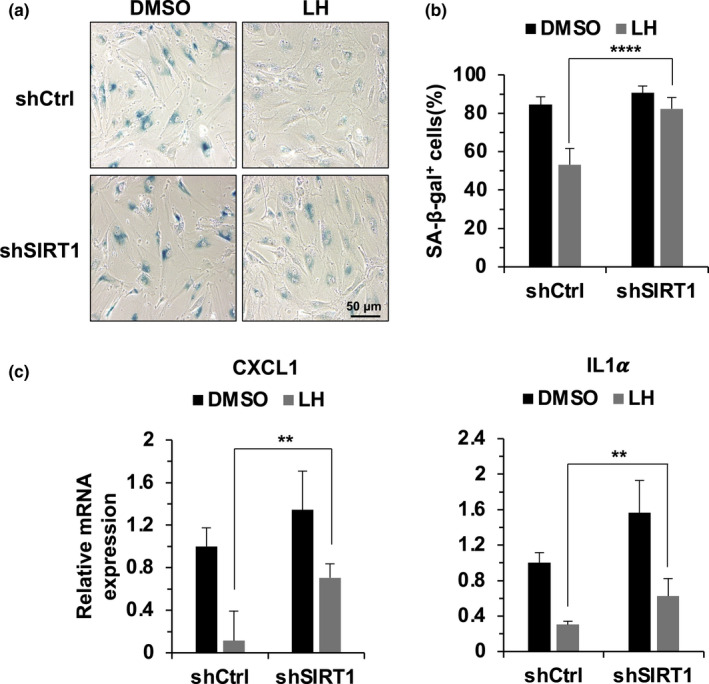
SIRT1 depletion abrogates the lycorine hydrochloride‐mediated suppression of the onset of SIPS and the expression of SASP factors. (a,b) Knocking down SIRT1 abrogated the lycorine hydrochloride‐mediated suppression of the onset of SIPS. The procedure for the induction of SIPS is described in Figure 1. SIRT1‐depleted cells were treated with lycorine hydrochloride one day before X‐ray irradiation. SA‐β‐gal staining was performed on day 14 post‐IR, followed by the quantification of SA‐β‐gal‐positive cells. (c) Lycorine hydrochloride‐induced suppression of SASP factor expression was partially abrogated by SIRT1 depletion. SIRT1‐depleted cells were treated with lycorine hydrochloride (1 μM) for one day before X‐ray irradiation. The cells were then collected on day 6 post‐IR. The relative mRNA expression of the indicated SASP factors was analyzed by quantitative RT‐PCR. Error bars represent SD **p < 0.01, ****p < 0.001, *t* test. All experiments were repeated at least three times. LH, lycorine hydrochloride.

Importantly, knocking down SIRT1 in HCA2‐hTERT cells abrogated the lycorine hydrochloride‐mediated onset of SIPS (Figure 5a,b). As a consequence, the decline in SASP expression was also rescued in lycorine hydrochloride‐treated SIRT1‐depleted cells (Figure 5c). Similar results were observed in cells treated with EX527 (Figure S7c–e).

Taken together, our results show that by promoting the expression of SIRT1, lycorine hydrochloride activates DNA repair by both NHEJ and HR and stabilizes the genomes, thereby suppressing the onset of SIPS and the expression of associated SASP factors.

## 3 DISCUSSION

A number of senolytic drugs have been developed to selectively induce the apoptosis of senescent cells in aging tissues (Chang et al., 2016; Zhu et al., 2015, 2016). These drugs have shown great potential in delaying the onset of aging and age‐associated diseases (Xu et al., 2018; Yousefzadeh et al., 2018). However, drugs that target DNA repair to block the onset of SIPS remain largely undeveloped. Here, we found that lycorine hydrochloride, which exhibits an anti‐AD function by targeting acetylcholinesterase (AChE), promotes DNA repair by both HR and NHEJ, thereby inhibiting SIPS and the expression of the SASP factors. This finding suggests that, in addition to developing drugs to eliminate senescent cells, finding drugs to delay the onset of senescence to combat aging might be an effective approach.

Several small molecules have been developed to target DNA repair pathways. For instance, RS‐1 activates RAD51 to promote HR repair (Mason et al., 2014), while nicorandil targets APE1 to activate the base excision repair (BER) pathway (Georgiadis et al., 2016). In addition, aspirin is capable of increasing life span by affecting mismatch repair (MMR) (McIlhatton et al., 2011). Our recent work demonstrates that farrerol promotes HR repair by facilitating the recruitment of RAD51, while it has no impact on the NHEJ pathway (Zhang et al., 2020). However, whether these small molecules can be used to suppress senescence *in vitro* and improve health span or life span has not been studied. Our work on lycorine hydrochloride expands the list of small molecules promoting DNA repair, but a detailed and thorough *in vivo* study on its effect on aging and aging‐related diseases is warranted. Moreover, since lycorine hydrochloride has multiple targets (Cao et al., 2013; Ji et al., 2017), whether it regulates healthspan or life span and whether it directly targets DNA repair to improve health span or life span needs to be clarified.

In this study, we demonstrated that lycorine hydrochloride promoted the expression of SIRT1 and SIRT6, critical longevity genes, at the transcriptional level, thereby activating both the HR and NHEJ pathways. The upregulation of these two genes might indicate a hormetic response, as lycorine hydrochloride at high concentrations blocks protein synthesis (Baez & Vazquez, 1978), inhibits cell proliferation and induces apoptosis (Cao et al., 2013). Although SIRT6 expression is only mildly promoted, it participates in the regulation of both HR and NHEJ repair (Chen et al., 2017; Mao et al., 2011) and the senescence of different types of cells, such as chondrocytes (Nagai et al., 2015) and endothelial cells (Liu et al., 2014). Thus, the effect of lycorine hydrochloride on the promotion of DSB repair and the delay of cellular senescence might be partly attributable to the upregulation of SIRT6. However, the regulatory mechanisms by which these two sirtuins are stimulated need to be further elucidated.

Our study indicates that lycorine hydrochloride induces low toxicity in HCA2 fibroblasts, but previous studies have shown that a high concentration of lycorine hydrochloride might induce cell cycle arrest in cancer cell lines (Cao et al., 2013). Before clinical application, the toxicity to animals and the pharmacokinetic properties need to be further examined.

In summary, we demonstrated, for the first time, that lycorine hydrochloride promotes DSB repair by both HR and NHEJ by enhancing the expression of SIRT1 at the transcriptional level, thereby suppressing the onset of SIPS and the associated SASP. Our work may encourage other researchers to identify novel small molecules to promote DNA repair, therefore delaying the onset of aging and aging‐associated diseases.

## 4 MATERIALS AND METHODS

### 4.1 Cell culture

HCA2‐H15c and HCA2‐I7c cell lines are derived from HCA2‐hTERT (Mao et al., 2008), which are two report cassettes used to detect HR and NHEJ repair efficiency, respectively. All fibroblast lines were cultured in DMEM (Sigma) supplemented with 10% fetal bovine serum (Gibco, Cat. # 10270‐106), 1% NEAA (Gibco, Cat. # 11140‐050) and 1% penicillin/streptomycin (Gibco, Cat. # 15140‐122). All cells were maintained at 37°C in a 5% CO_2_ atmosphere.

### 4.2 Transfection and lycorine hydrochloride treatment

All HCA2‐hTERT‐derived cell lines were electroporated with the indicated amount of DNA using a Lonza 4D machine with the DT‐130 program. For the measurement of NHEJ and HR repair efficiency, 5 μg of the I‐SceI vector and 15 ng of the DsRed were transfected into reporter cells (HCA2‐I7c or HCA2‐H15c) pretreated with lycorine hydrochloride or/and EX527. The transfected cell medium was supplemented with small molecules at the indicated concentrations until a FACS analysis was performed 3 days posttransfection. Both lycorine hydrochloride and EX527 were purchased from Selleck (lycorine hydrochloride, S3800; EX527, S1541).

### 4.3 Plasmids and antibodies

Viral vectors bearing shRNAs were generated based on the pLKO1 vector. The sequence targeting SIRT1 was 5′‐CATGAAGTGCCTCAGATATTA‐3′. For the luciferase reporters, the promoters of SIRT1 and SIRT6 were amplified from genomic DNA of HCA2‐hTERT cells and cloned into a pGL3‐basic plasmid.

The antibodies used in the study were as follows: anti‐53BP1 (CST, Cat. # 4937S), anti‐DNA‐PKcs (Abcam, Cat. # ab32566), anti‐RAD50 (Abclonal, Cat. # A3078), anti‐NBS1 (CST, Cat. # 3002), anti‐BRCA1 (Abclonal, Cat. # A0212), anti‐CtIP (active motif, Cat. # 61141), anti‐KU70 (Abclonal, Cat. # A0883), anti‐KU80 (Abclonal, Cat. # A5862), anti‐LIG4 (Abclonal, Cat. # A1743), anti‐MRE11 (Abclonal, Cat. # A2559), anti‐PARP1 (Abclonal, Cat. # A3121), anti‐XLF (Abclonal, Cat. # A4985), anti‐XRCC4 (Abclonal, Cat. # A7539), anti‐β‐tubulin (CMCTAG, Cat. # AT0050), anti‐EXO1 (Abclonal, Cat. # A6810), anti‐RPA2 (Abclonal, Cat. # A2189), anti‐RAD51 (Abclonal, Cat. # ab88572), anti‐SIRT1 (Abcam, Cat. # ab110304), anti‐SIRT2 (Abclonal, Cat # A0273), anti‐SIRT6 (Abcam, Cat. # ab62738), anti‐SIRT7 (Proteintech, Cat. # 12994‐1‐AP), anti‐p16 (Abcam, Cat. # ab108349), and anti‐p21 (Abcam, Cat. # ab109199).

### 4.4 Luciferase assay

HCA2 cells were seeded at a density of 5 × 10^5^ cells per plate and incubated for 48 h before being transfected with the luciferase reporters. The cells were collected and lysed 48 h posttransfection. Luciferase activities were measured by a dual‐luciferase reporter system (Promega, Cat. #E1910) and a GloMax Luminometer (Promega, Cat. #E5311). All luciferase assays were performed at least three times.

### 4.5 Western blot analysis

Forty‐eight hours after lycorine hydrochloride treatment, cells were harvested for protein extraction. The cells were lysed in SDS lysis buffer (1% SDS; 10 mM EDTA; 50 mM Tris‐HCl, pH 8.1; and protease inhibitors). After protein quantification and normalization, equivalent amounts of proteins were electrophoresed on 8–12% SDS‐PAGE gels followed by Western blot analysis.

### 4.6 Immunofluorescence

Cells were seeded on slides incubated with lycorine hydrochloride for 24 h. Then, the cells were washed twice with PBS and fixed with 4% paraformaldehyde for 15 min at room temperature, followed by washing with PBS three times. The fixed cells were permeabilized with 0.25% Triton X‐100 for 10 min and washed with PBS three times. Then, the cells were blocked with 1% BSA for 1 h at room temperature. Next, the cells were incubated overnight with anti‐γH2AX (Cell Signaling Technology, Cat. #9718S), anti‐53BP1 (Cell Signaling Technology, Cat. #4937S) or anti‐RPA2 antibody diluted to 1:200 in 1% BSA at 4°C. After being washed three times with cold PBS, the cells were incubated with FITC‐conjugated secondary antibodies (Abcam, goat‐anti‐rabbit‐FITC, Cat. #ab6717) for 1 h at room temperature in the dark, followed by treatment with 1 mg/mL DAPI (Abcam, Cat. #ab104939) staining. Pictures of stained cells were taken on a Nikon A1R laser scanning confocal microscope.

### 4.7 Quantitative RT‐PCR

Total RNA was extracted using a commercial kit (TIANGEN) according to the manufacturer's instructions. Real‐time PCR was performed using the Roche Universal Probe Library (UPL) system on an ABI according to the manufacturer's specifications. The primer sequences were referenced by (Laberge et al., 2015).

### 4.8 Cell proliferation

Cells were seeded at the same density and treated with the indicated concentration of lycorine hydrochloride and harvested at different time points after seeding to enable a cell count.

### 4.9 Cell cycle

Cell cycle distribution was examined by PI staining assay. Briefly, cells were harvested and fixed with cold 70% ethanol for at least 16 h. After fixation, the cells were washed twice with PBS, followed by incubation with 1 mL of PBS containing 20 μg/mL PI and 1 mg/mL RNase A for 30 min at room temperature. The PI stained samples were analyzed by FACS. At least 10,000 events were included in each analysis. The data were analyzed by FlowJo software.

### 4.10 Apoptosis

Apoptosis caused by lycorine hydrochloride was analyzed by an Annexin‐V‐FITC apoptosis detection kit (BD Pharmingen). Briefly, cells (1–6 × 10^5^ per sample) were collected, washed twice with cold PBS, centrifuged, and resuspended in 100 μL of binding buffer. Then, Annexin‐V‐FITC and PI staining solution were added. After incubation for 10 min in the dark, the fluorescence was analyzed using an FITC Annexin‐V apoptosis detection kit.

### 4.11 β‐Gal staining

To induce senescence by IR, cells were incubated with lycorine hydrochloride and then irradiated with X‐rays generated by an RS2000Pro source. Ten to fourteen days later, the cells were fixed in 2% formaldehyde and 0.2% glutaraldehyde in PBS for 5 min at room temperature and washed twice with PBS. Then, staining solution (1 mg/mL X‐gal in dimethylformamide, 40 mM citric acid/sodium phosphate buffer, 5 mM potassium ferrocyanide, 5 mM potassium ferricyanide, 150 mM sodium chloride, and 2 mM magnesium chloride) was added, and the cells were incubated for 16 h at 37°C. Images of the stained cells were taken with a bright‐field microscope.

### 4.12 Comet assay

Cells were seeded at a density of 2 × 10^4^ cells per well on 6‐well plates and treated with compound for 24 h. On day 3, the cells were irradiated by X‐ray at 8 Gy, followed by comet assay analysis 4 h post‐IR. The detailed procedure is as described in the manufacturer's instructions (Trevigen, Cat. # 4250–050‐K).

## CONFLICT OF INTEREST

The authors declare that they have no competing interests.

## AUTHOR CONTRIBUTIONS

YJ, WL, RX and ZM designed, coordinated, and oversaw the study. WZ and JY designed and conducted the experiments and performed the statistical analysis. YC provided helpful advice and feedback on various aspects of the study design. WZ, JY, WL, YJ, and ZM wrote the paper. All authors contributed to, critically reviewed, and approved the manuscript.

## Supporting information

Figures S1–S7

Figure Legend

## Data Availability

Data sharing is not applicable to this article as no new data were created or analyzed in this study.
