# Supplementary material for: Lycorine hydrochloride suppresses stress‐induced premature cellular senescence by stabilizing the genome of human cells
Source: Aging Cell. 2021 Jan 17;20(2):e13307. doi: 10.1111/acel.13307 (PMC7884038; doi:10.1111/acel.13307)
Supplement: Supplementary file 1 — Figures S1–S7 [file ACEL-20-e13307-s001.pdf]

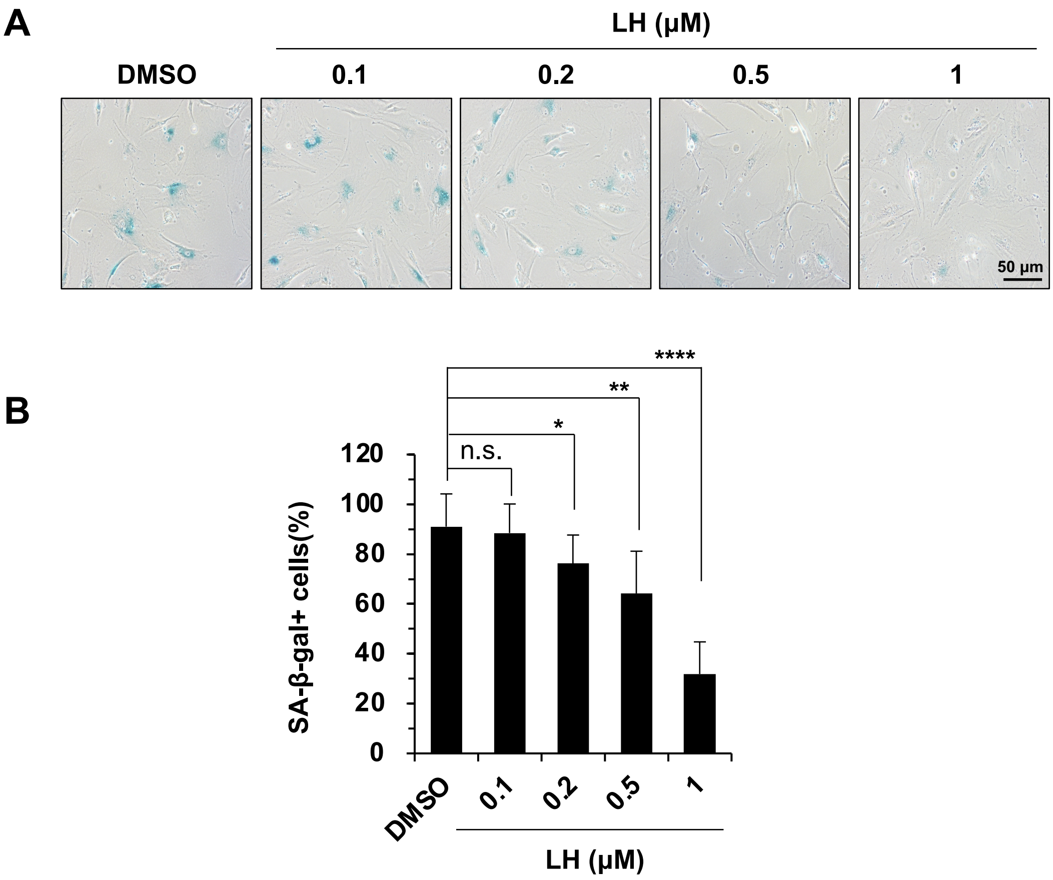

Supplementary Figure 1

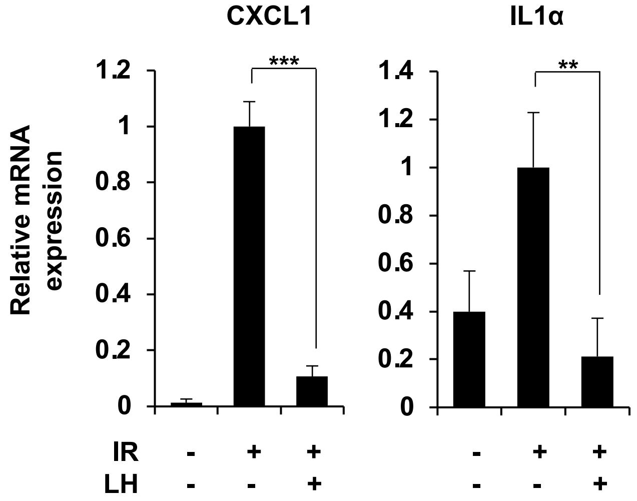

Supplementary Figure 2

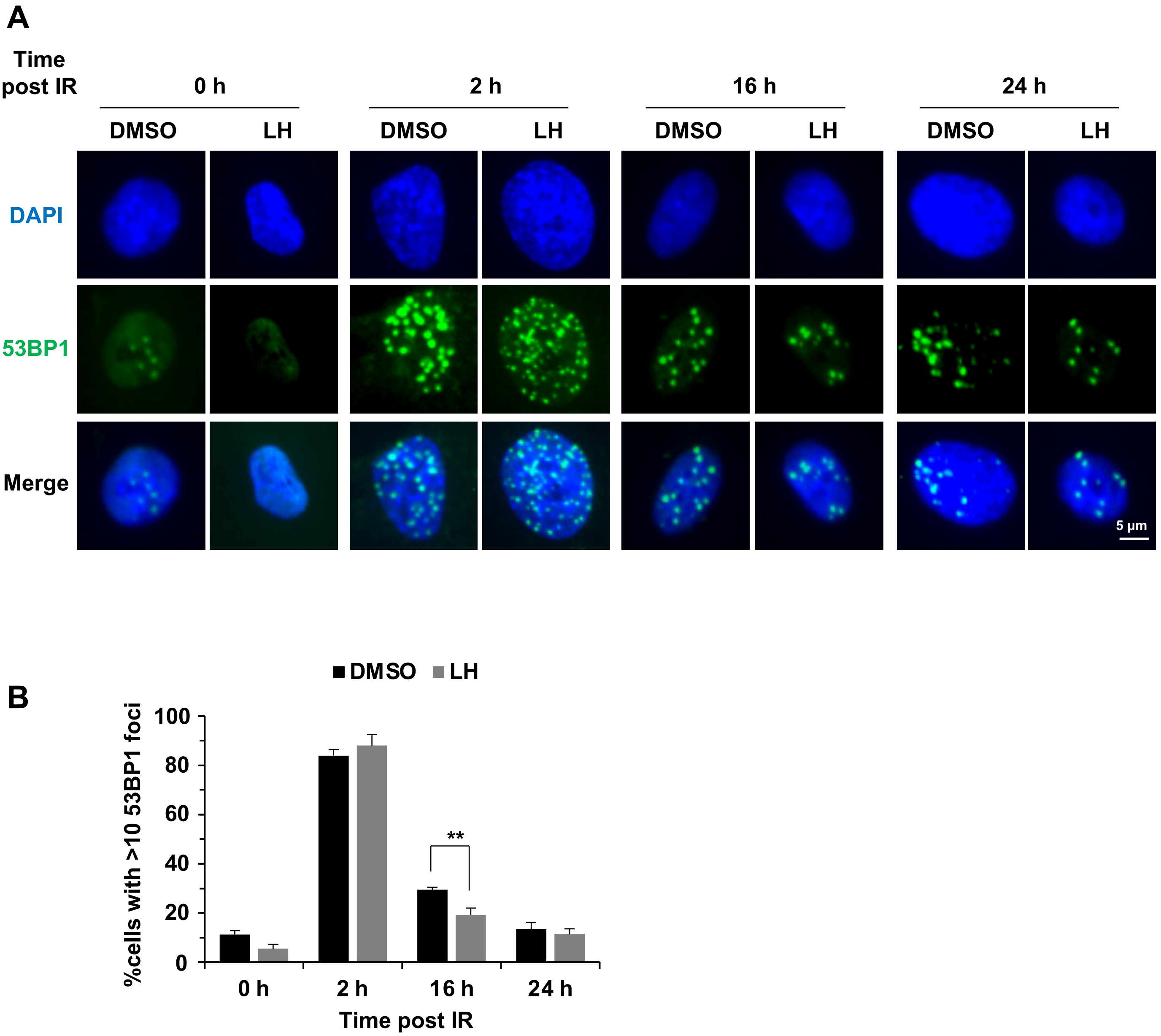

Supplementary Figure 3

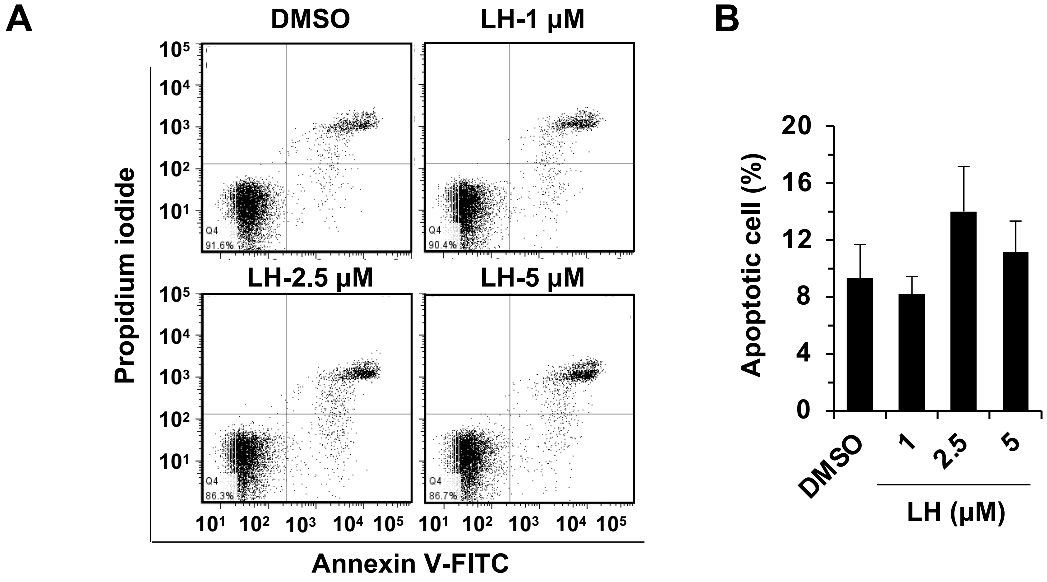

Supplementary Figure 4

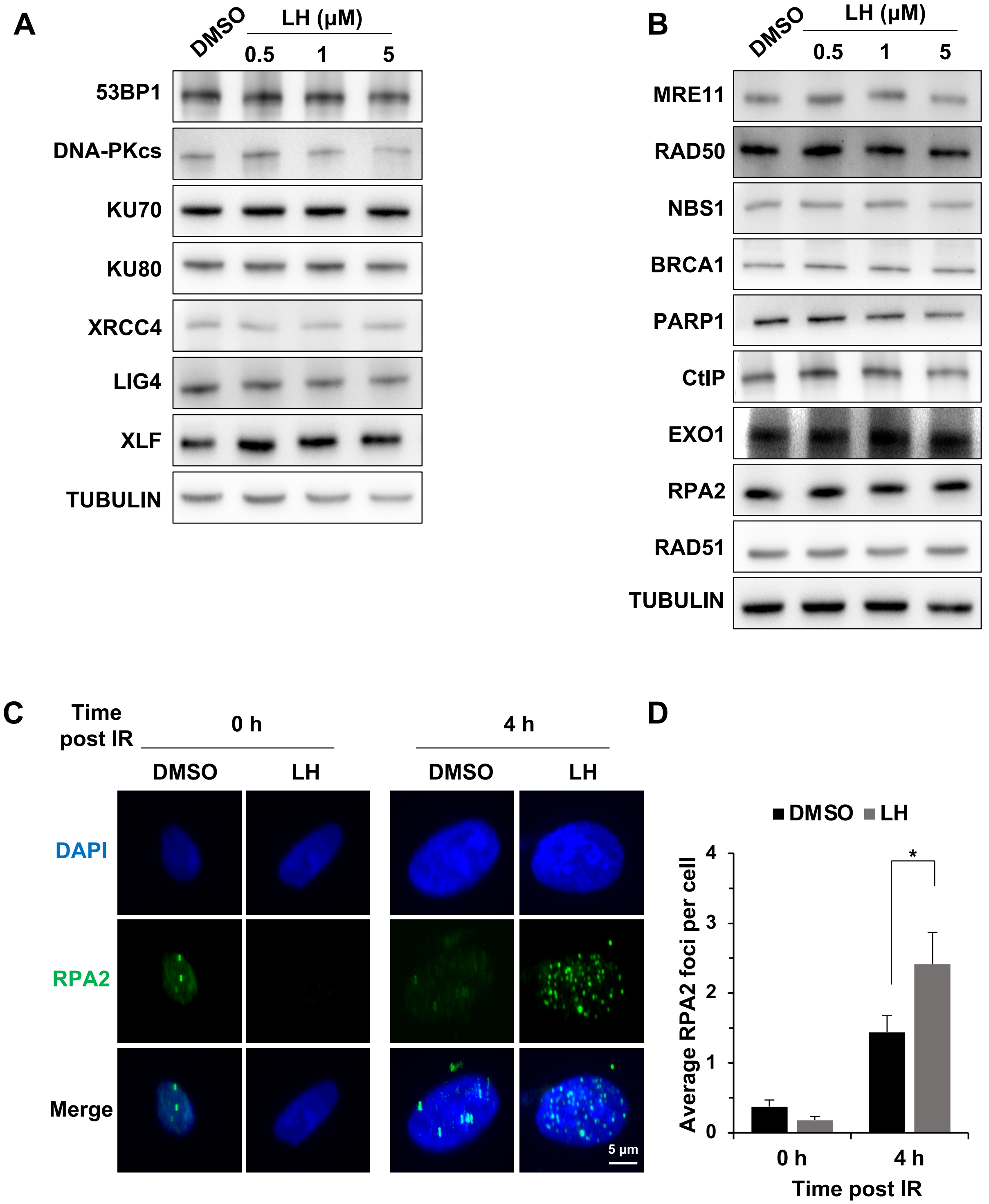

Supplementary Figure 5

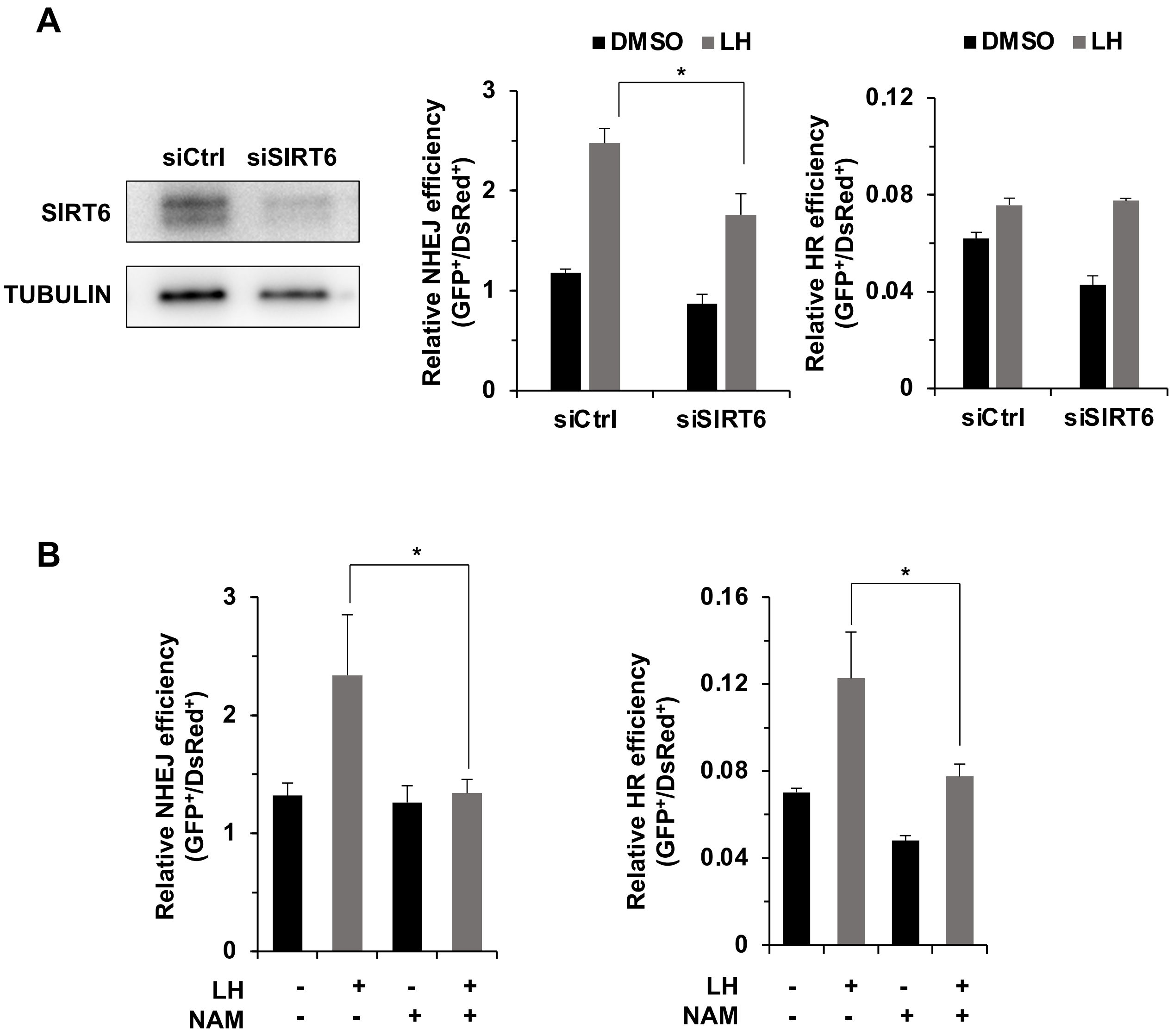

Supplementary Figure 6

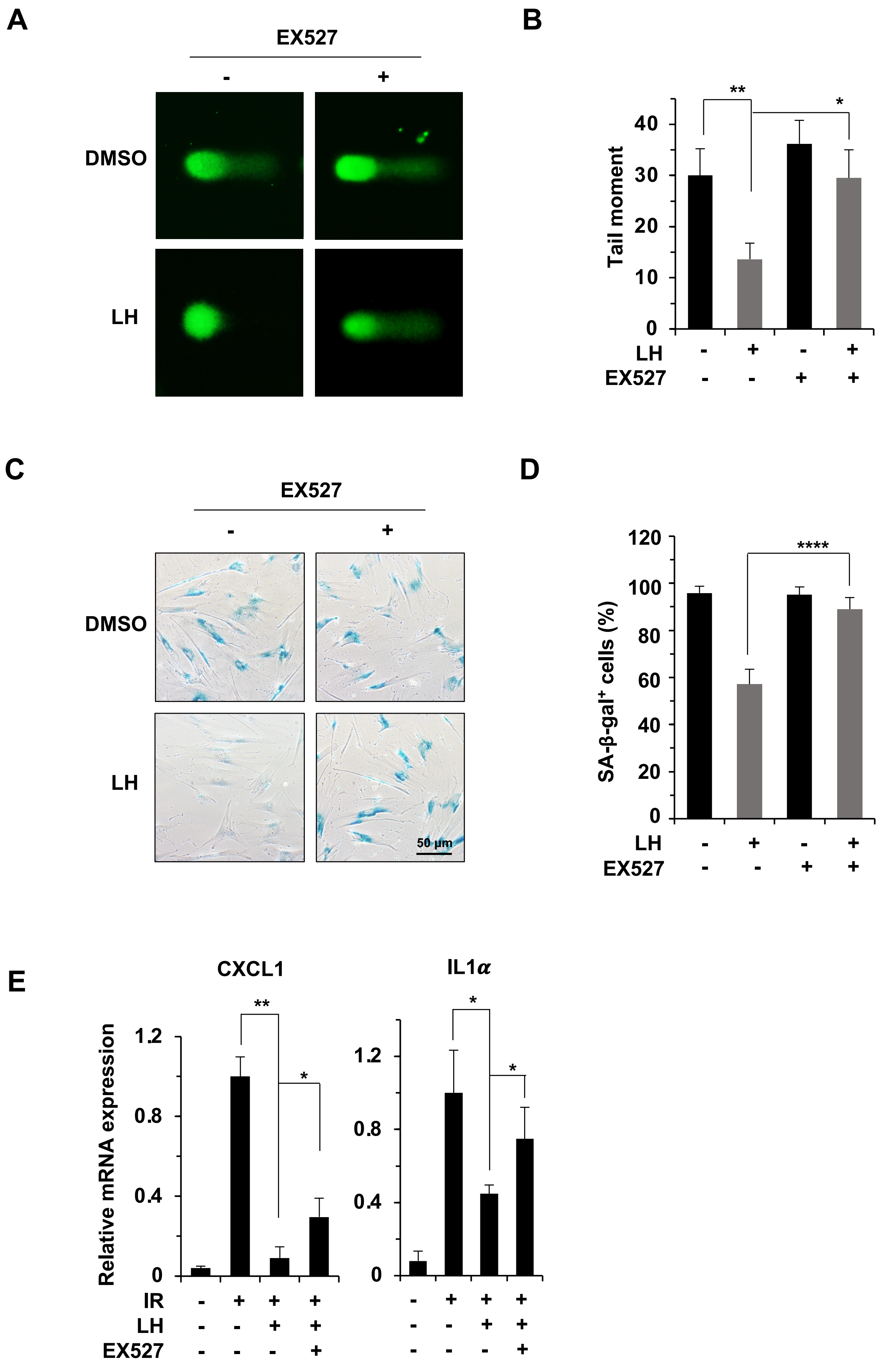

Supplementary Figure 7
