# Supplementary material for: Lycorine hydrochloride suppresses stress‐induced premature cellular senescence by stabilizing the genome of human cells
Source: Aging Cell. 2021 Jan 17;20(2):e13307. doi: 10.1111/acel.13307 (PMC7884038; doi:10.1111/acel.13307)
Supplement: Supplementary file 2 — Figure Legend [file ACEL-20-e13307-s002.docx]

**Supplementary figure legends**

**Supplementary Figure 1. Lycorine hydrochloride suppresses the onset of cellular senescence in a dose-dependent manner.**

(A-B) HCA2 cells were pretreated with lycorine hydrochloride at the indicated concentrations one day before X-ray (10 Gy), and the cells were harvested on day 10 postirradiation and then stained for SA-β-gal detection. Representative images are shown in (A), and the percentage of SA-β-gal-positive cells is shown in (B). Error bars represent the s.e.m. * *P* < 0.05, ** *P* < 0.01, **** *P* < 0.001, t-test. LH, lycorine hydrochloride.

**Supplementary Figure 2. Pretreating SIPS cells with lycorine hydrochloride affects the expression of SASP factors.**

HCA2 cells were exposed to X-rays at a dosage of 50 Gy to obtain senescent cells, which were incubated with lycorine hydrochloride at a concentration of 1 μM for 5 days before the analysis of mRNA expression. Error bars represent s.d. ** *P* < 0.01, *** *P* < 0.005, t-test. All experiments were repeated at least three times. LH, lycorine hydrochloride.

**Supplementary Figure 3. Lycorine hydrochloride accelerates the clearance of 53BP1 foci.**

(A-B) HCA2 cells were exposed to X-rays at a dosage of 2 Gy and incubated with lycorine hydrochloride at a concentration of 1 μM for 24 h, and then, immunofluorescence experiments were performed to quantify the 53BP1 foci-positive cells at the indicated time points. Representative images are shown in (A), and the percentage of cells with > 10 53BP1 foci is shown in (B). Error bars represent the s.e.m. ** *P* < 0.01, t-test. All experiments were repeated at least three times. LH, lycorine hydrochloride.

**Supplementary Figure 4. The effects of lycorine hydrochloride on apoptosis.**

(A-B) HCA2 cells were treated with lycorine hydrochloride at the indicated concentration for 48 h and then, stained by Annexin V/PI staining to analyze the apoptotic rates by FACS. Error bars represent s.d. The experiment was repeated at least three times. LH, lycorine hydrochloride.

**Supplementary Figure 5. Lycorine hydrochloride has no effect on the expression of proteins involved in DSB repair but accelerates the recruitment of RPA2.**

(A-B) Lycorine hydrochloride does not influence the protein expression of important NHEJ (A) and HR (B) pathway-associated factors. (C-D) Lycorine hydrochloride promotes the recruitment of RPA2. Lycorine hydrochloride (1 μM)-pretreated HCA2 cells were irradiated at a dose of 2 Gy and then immunostained to quantify the number of RPA2 foci-positive cells at the indicated time points. Representative images are shown in (C), and the quantification of RPA2 foci numbers is shown in (D). At least 50 cells were included for each group. Error bars represent the s.e.m. * *P* < 0.05, t-test. All experiments were repeated at least three times. LH, lycorine hydrochloride.

**Supplementary Figure 6. Lycorine hydrochloride-mediated stimulation of DSB repair is partially dependent on SIRT6.**

(A) Western blot analysis of SIRT6 depletion in HCA2-hTERT cells with siRNA targeting the SIRT6 gene (left panel). Knocking down SIRT6 impaired the lycorine hydrochloride-mediated promotion of NHEJ but not HR repair (middle and right panel). HCA2-hTERT cells harboring NHEJ or HR reporters were transfected twice with siRNA targeting SIRT6 with a two-day interval, and during the second siRNA transfection, vectors encoding I-SceI and DsRed were cotransfected into the cells. Lycorine hydrochloride at a concentration of 1 μM was added to the transfected cells immediately after the second transfection. On day 3, the cells were harvested and analyzed by FACS. (B) Inhibiting SIRT1 and SIRT6 activity with nicotinamide (NAM) abrogated the promotion of lycorine hydrochloride on DNA repair efficiency. NAM at a final concentration of 5 mM was added to the cells 24 h before transfection and maintained in the culture after the transfection of the I-SceI vectors and DsRed vectors. Error bars represent s.d. * *P* < 0.05, t-test. All experiments were repeated at least three times. LH, lycorine hydrochloride.

**Supplementary Figure 7. Blocking** **SIRT1 enzyme activity abrogates the lycorine hydrochloride-mediated effects on genomic stability, the onset of SIPS and the expression of SASP factors.**

(A) Blocking SIRT1 enzyme activity with EX527, a SIRT1 inhibitor, abrogated the promotion effect of lycorine hydrochloride on genomic stability. Representative images of the alkaline comet assay used for analyzing the genomic stability of HCA2 cells treated with 1 μM lycorine hydrochloride in the presence or absence of EX527. The working concentration of EX527 was 20 μM. (B) Quantification of the tail moment from at least 50 cells. (C-D) Inhibiting SIRT1 enzyme activity abrogated the lycorine hydrochloride-mediated suppression of the onset of SIPS. The procedure of the induction of SIPS is described in Figure 1. Lycorine hydrochloride was added to HCA2 cells one day before X-ray irradiation. SA-β-gal staining was performed on day 14 post-IR, and then, the SA-β-gal-positive cells were quantified. Representative images are shown in (C), and the percentage of SA-β-gal-positive cells is shown in (D). (E) Lycorine hydrochloride-induced suppression of SASP factor expression was partially abrogated by inhibiting SIRT1 enzyme activity. HCA2 cells were treated with lycorine hydrochloride and EX527 one day before X-ray irradiation. The cells were then collected on day 6 post-IR. Relative mRNA expression of the indicated SASP factors was analyzed by quantitative RT-PCR. Error bars represent s.d. * *P* < 0.05, ** *P* < 0.01, **** *P* < 0.001, t-test. All experiments were repeated at least three times. LH, lycorine hydrochloride.
